# A Novel Regulator PepR Regulates the Expression of Dipeptidase Gene *pepV* in *Bacillus thuringiensis*

**DOI:** 10.3390/microorganisms12030579

**Published:** 2024-03-14

**Authors:** Xin Zhang, Hengjie Wang, Tinglu Yan, Yuhan Chen, Qi Peng, Fuping Song

**Affiliations:** 1 State Key Laboratory for Biology of Plant Diseases and Insect Pests, Institute of Plant Protection, Chinese Academy of Agricultural Sciences, Beijing 100193, China; zhangxin_live@outlook.com (X.Z.); whj18911651571@163.com (H.W.); yantinglu9969@163.com (T.Y.); chenyuhan0909@163.com (Y.C.); qpeng@ippcaas.cn (Q.P.); 2 College of Life Science, Northeast Agricultural University, Harbin 150030, China

**Keywords:** transcriptional regulator PepR, dipeptidase gene *pepV*, resistant vancomycin resistance, *Bacillus thuringiensis*

## Abstract

*Bacillus thuringiensis* produces insecticidal crystal proteins encoded by *cry* or *cyt* genes and targets a variety of insect pests. We previously found that a strong promoter of a DeoR family transcriptional regulator (HD73_5014) can efficiently drive *cry1Ac* expression in *B. thuringiensis* HD73. Here, we investigated the regulation of neighbor genes by HD73_5014. The HD73_5014 homologs are widely distributed in Gram-positive bacterial species. Its neighbor genes include *pepV*, *rsuA*, and *ytgP*, which encode dipeptidase, rRNA pseudouridine synthase and polysaccharide biosynthesis protein, respectively. The four open reading frames (ORFs) are organized to be a *pepR* gene cluster in HD73. RT-PCR analysis revealed that the *rsuA* and *ytgP* genes formed a transcriptional unit (*rsuA*-*ytgP* operon), while *pepV* formed a transcriptional unit in HD73. Promoter-*lacZ* fusion assays showed that the *pepV* and *rsuA*-*ytgP* promoters are regulated by HD73_5014. EMSA experiments showed that HD73_5014 directly binds to the *pepV* promoter region but not to the *rusA*-*ytgP* promoter region. Thus, the HD73_5014 transcriptional regulator, which controls the expression of the dipeptidase *pepV*, was named PepR (dipeptidase regulator). We also confirmed the direct regulation between PepR and PepV by the increased sensitivity to vancomycin in Δ*pepV* and Δ*pepR* mutants compared to HD73.

## 1. Introduction

*Bacillus thuringiensis* (Bt) is a very important microorganism in the biological control of plant pests and diseases [1]. During sporulation, it can produce crystals which are mainly formed by the insecticidal crystal proteins and are toxic to many pests, including more than 500 species in nine orders such as *Lepidoptera*, *Hymenoptera*, *Diptera*, *Coleoptera*, *Trichoptera*, *Orthoptera*, and so on [2]. As of an update dated 22 February 2024, approximately 858 *cry* and *cyt* genes encoding crystal proteins have been discovered (http://www.lifesci.sussex.ac.uk/home/Neil_Crickmore/Bt/). The *cry* genes express during the stationary phase, and crystal production constitutes 20 to 30% of the dry weight of sporulating cells [3].

Several *cry* and non-*cry* gene promoters have been identified to effectively direct *cry* gene expression. For example, the *cry1Ac* promoter can direct the expression of a variety of *cry*-like genes [4], such as *cry1Ac*, *cry1Ab*, *cry1Ac5*, *cry1Ac*-*av3*, *cry2Ab27*, and *cry8*. Similarly, the expression of Cry1AbMod/Cry1AcMod [5], Cry1Ac [6], Cry1c [7], Cry3A [8], Cry8Ga [9], and Cry69Aa1 [10] can be directed by the *cry3A* promoter. Recent reports have shown that some non-*cry* gene promoters with high-level activity were utilized for the expression of *cry* genes [11,12]. P*exsY* is a strong promoter of the exosporium basal layer structural gene *exsY* in late sporulation which has been used to express *cry1Ac* genes in Bt [11]. P*5014*, a non-*cry* gene strong promoter controlled by Sigma E, was used to strongly direct *cry1Ac* expression in Bt HD73. The *HD73_5014* gene with promoter P*5014* was annotated as the DeoR family transcriptional regulator [12]. However, the function and the target gene of HD73_5014 as transcriptional regulator are still unknown.

In the HD73 genome, the upstream of the *HD73_5014* gene was the *pepV* gene, which encodes a dipeptidase. Dipeptidases are involved in the final breakdown of protein degradation fragments produced by other peptidases (e.g., aminopeptidasesare PepN, PepC, PepP, PepX, PepA [13] and endopeptidasesare PepO1, PepO2, PepF1 and PepF2 [14,15]). In *Lactococcus lactis* MG1363, PepV has been found to be involved in resistance to vancomycin. The transcription of *pepV* in *L. helveticus* was regulated by a CodY-like regulator and the BCAA-responsive transcriptional regulator which binds adjacent to the *pepV* promoter region [16,17]. However, whether PepV had a similar function and its transcription regulated by PepR (HD73_5014, encoding dipeptidase regulator) required further investigation in *B. thuringiensis*.

## 2. Materials and Methods

### 2.1. Bacterial Strains, Plasmids, and Growth Conditions

*Bacillus thuringiensis* HD73 and its derivatives were cultured at 30 °C in Luria–Bertani (LB) medium (1% tryptone, 0.5% yeast extract, and 0.5% NaCl) or on solid LB medium supplemented with 1.5% agar. Schaeffer’s sporulation medium [18] (SSM; 0.8% nutrient broth, 0.012% MgSO_4_, 0.1% KCl, 0.5 mM NaOH, 1 mM Ca(NO_3_)_2_, 0.01 μM MnCl_2_, and 1 μM FeSO_4_) was used to observe the development of bacterial cells. *Escherichia coli* TG1 was used for molecular cloning experiments, and *E. coli* ET 12567 was used for producing non-methylated plasmid DNA for *B. thuringiensis* transformations [19,20]. These strains were cultured at 37 °C in LB medium. When required, antibiotics were added at the following concentrations for growth of *B. thuringiensis*: 5 μg/mL erythromycin, 50 μg/mL kanamycin, and 6 ng/mL vancomycin. For growth of *E. coli*, 100 μg/mL ampicillin was added. The bacterial strains and plasmids used in this study are summarized in Table 1.

### 2.2. DNA Manipulation and Transformation

Plasmid DNA was extracted from *E. coli* cells with a Plasmid Miniprep Kit (Axygen, Beijing, China). Restriction enzymes and T4 DNA ligase (Takara Biotechnology Corporation, Dalian, China) were used according to the manufacturer’s instructions. PCR was performed with high-fidelity PrimeSTAR HS DNA polymerase (Takara Biotechnology Corporation, Dalian, China) or *Taq* DNA polymerase (BioMed, Beijing, China). DNA fragments were purified from 1% agarose gels using an AxyPrep DNA Gel Extraction Kit (Axygen). Standard procedures were followed for *E. coli* transformation [25], and Bt HD73 cells were transformed by electroporation, as previously described [26].

### 2.3. RNA Isolation and Reverse Transcription PCR (RT-PCR) of pepR Neighbor Genes

The HD73 strain was cultured in SSM at 30 °C and harvested at T_6_. Total RNA was extracted using the RNAprep Pure Bacteria Kit (Aidlab, Beijing, China). The RNA (500 ng) was used for reverse transcription using the HiScript III 1st Strand cDNA Synthesis Kit (Vazyme, Nanjing, China). The primers were used to detect the expression of *pepR* and neighbor genes locus, as shown in Table 2. To confirm the absence of DNA contamination, all RNA samples were routinely subjected to 16S rRNA gene PCR using 16S rRNA-F/16S rRNA-R primers.

### 2.4. β-Galactosidase Activity Assays

The *pepV* (P*pepV*, 360 bp) and *rsuA*-*ytgP* (P*rsuA*-*ytgP*, 402 bp) promoters were amplified from HD73 genomic DNA using primers P*pepV*-F/P*pepV*-R and P*rsuA*-*ytgP*-F/P*rsuA*-*ytgP*-R, respectively. P*pepV* and P*rsuA*-*ytgP* were digested with PstI and BamHI sites, followed by ligation into the linearized pHT304-18Z plasmid, which harbors a promoterless *lacZ* [22], to obtain the recombinant plasmid, 304P*pepV* and 304P*rsuA*-*ytgP*. The 304P*pepV* and 304P*rsuA*-*ytgP* plasmid were introduced into HD73 and ∆*pepR*, resulting in HD (P*pepV*), ∆*pepR* (P*pepV*), HD (P*rsuA*-*ytgP*), and ∆*pepR* (P*rsuA*-*ytgP*), respectively. The HD (P*pepV*), ∆*pepR* (P*pepV*), HD (P*rsuA*-*ytgP*), and ∆*pepR* (P*rsuA*-*ytgP*) strains were validated by erythromycin and PCR.

To detect the transcriptional activity of the P*pepV* and P*rsuA*-*ytgP* promoters in HD73 and Δ*pepR* strains, HD (P*pepV*), ∆*pepR* (P*pepV*), HD (P*rsuA*-*ytgP*), and ∆*pepR* (P*rsuA*-*ytgP*) were cultured in SSM medium at 30 °C with shaking 220 rpm. Two milliliters of culture were collected at 1 h intervals from T_0_ to T_7_ (T_0_ indicates the end of the exponential growth phase and T_n_ indicates n hours after time T_0_). The cells were centrifuged (12,000× *g*, 1 min), and the pellets were stored at −20 °C until use. β-galactosidase activities were measured as previously described [24]. Values are reported as the mean and standard error of at least three independent assays.

### 2.5. Expression and Purification of PepR

The pET*pepR* plasmid containing *pepR* from HD73 was constructed by amplifying *pepR* with primers pET*pepR*-F/pET*pepR*-R and cloning into BamHI/SalI-digested pET21b. The pET*pepR* was transferred into the *E. coli* BL21(DE3), and the positive transformants were BL21 (pET*pepR*). The transformants were grown to the OD_600nm_ of approximately 1.0 in LB medium supplemented with ampicillin; then, they were incubated with isopropyl-β-D-thiogalactoside (IPTG) at a final concentration of 1 mM in an 18 °C shaking incubator for 12 h. Total cell proteins from each optimization experiment were analyzed by sodium dodecyl sulfate–polyacrylamide gel electrophoresis (SDS-PAGE). The expression and purification of the PepR-His protein was performed as previously described [27].

### 2.6. Electrophoresis Mobility Shift Assays

The *pepV* fragment was PCR amplified from HD73 genomic DNA by specific primers labeled with a 5′-end FAM modification and confirmed by *pepV* sequencing. Electrophoresis mobility shift assays (EMSA) [28] were performed as described to analyze the binding of purified PepR.

### 2.7. Vancomycin Sensitivity Assay

The ∆*pepR*, ∆*pepV*, and HD73 strains were grown in LB medium until they reached an optical density of approximately 1.0 at 600 nm (OD 600 nm). Then, each strain suspension was divided into triplicates and transferred to 100 mL of SSM medium with a vancomycin concentration of 6 ng/mL. The OD 600 nm was measured every hour at 30 °C.

## 3. Results

### 3.1. PepR Homologs Are Widely Distributed in Various Gram-Positive Bacteria

**Figure 1:**
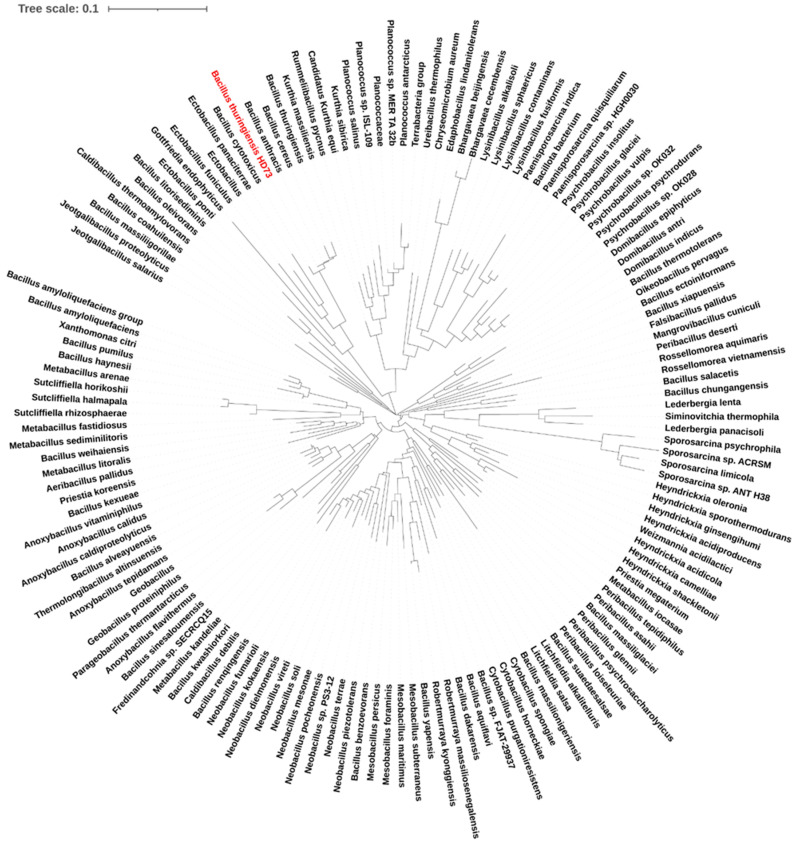
Evolution of PepR homologs in bacteria. Evolutionary analyses were conducted in MEGA7 using the neighbor-joining method. A total of 143 amino acid sequences were analyzed and an optimal tree was shown. Red text indicates the *B. thuringiensis* HD73 strain from which the reference sequence of the PepR homologs originated.

In a previous study, we found that the *pepR* gene promoter can efficiently direct *cry1Ac* expression in HD73 [12]. However, the function of the *pepR* gene and the target gene of PepR as a transcriptional regulator are still unknown. In this study, we analyzed PepR homologs through a phylogenetic evolutionary tree. PepR homologs with amino acid similarity greater than 70% and 100% amino acid coverage from the NCBI database were screened (Table S1). The PepR homologs were searched with an E-value lower than 2.07 × 10^−45^. The phylogenetic tree showed that the PepR homologs were present in 143 bacterial species, all of which were Gram-positive and mainly included *Anoxybacillus* spp., *Bacillus* spp., *Caldibacillus* spp., *Cytobacillus* spp., *Domibacillus* spp., *Ectobacillus* spp., *Heyndrickxia* spp., *Jeotgalibacillus* spp., *Kurthia* spp., *Lysinibacillus* spp., *Metabacillus* spp., *Neobacillus* spp., *Paenisporosarcina* spp., *Peribacillus* spp., *Planococcus* spp., *Psychrobacillus* spp., *Sporosarcina* spp., and *Sutcliffiella* spp., etc. (Figure 1). This finding suggests that PepR homologs are highly conserved across a wide range of Gram-positive bacteria.

### 3.2. Characterization of the Transcription Units in the pepR Gene Cluster

**Figure 2:**
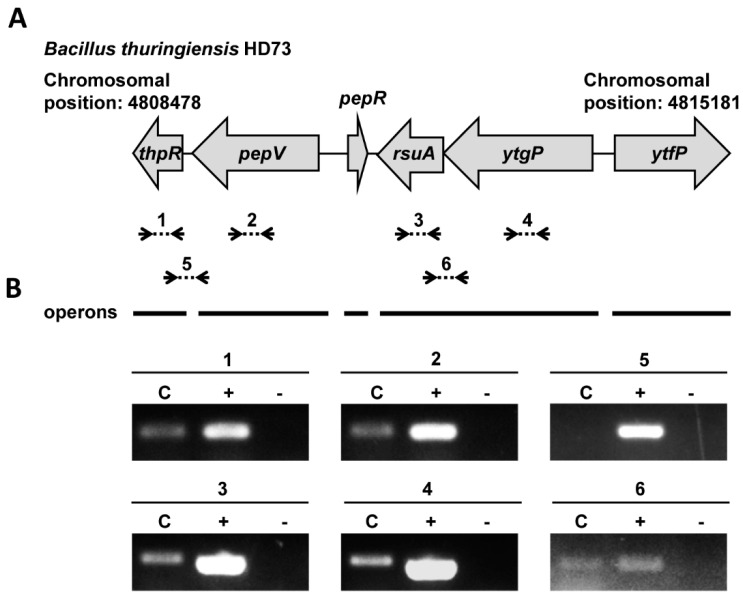
RT-PCR identified the *pepV* and *rsuA*-*ytgP* transcription units at the *pepR* gene locus in *B. thuringiensis* HD73. (**A**) Genetic organization of the *pepR* locus. Open reading frames (ORFs) are represented by grey arrows. The RT-PCR amplicons (see lanes in panel (**B**)) correspond to the dashed lines with small black arrows. The solid lines below the ORFs represent the operons. (**B**) RT-PCR analyzed the transcription units of *pepR* neighboring genes. RNA samples were prepared at T_6_ of bacterial culture in SSM medium. The RT-PCR labelled “C” was performed with 500 ng RNA. Positive controls are labelled “+” (PCR with 100 ng of genomic DNA). Negative controls are labelled “−” (RT-PCR with 500 ng RNA using heat-inactivated reverse transcriptase). Numbers represent different RT-PCR amplicons: numbers 1–4 represent *thpR*, *pepV*, *rsuA*, and *ytgP*; numbers 5 and 6 represent *thpR*-*pepV* and *rsuA*-*ytgP*, respectively.

The nucleotide sequence of the *pepR* gene cluster (4489 bp) of HD73 is comprised of four ORFs, which are annotated as dipeptidase (*pepV*, *HD73_5013*), DeoR family transcriptional regulator (*pepR*, *HD73*_*5014)*, rRNA pseudouridine synthase (*rsuA*, *HD73*_*5015*), and polysaccharide biosynthesis protein (*ytgP*, *HD73*_*5016*) (Figure 2A). To determine the transcription units of the *pepR* cluster, a series of primers were designed. The total RNA was extracted at T_6_ from *B. thuringiensis* HD73 cultures grown in SSM. RT-PCR results showed mRNA overlapping the *rsuA* and *ytgP* genes (Figure 2B). However, no mRNA overlapping *thpR* and *pepV* was transcribed. These results indicate that *rsuA* and *ytgP* genes form a transcriptional unit (*rsuA*-*ytgP* operon), while *thpR* and *pepV* each form a transcriptional unit independently.

### 3.3. PepR Positively Regulates pepV and rsuA-ytgP

**Figure 3:**
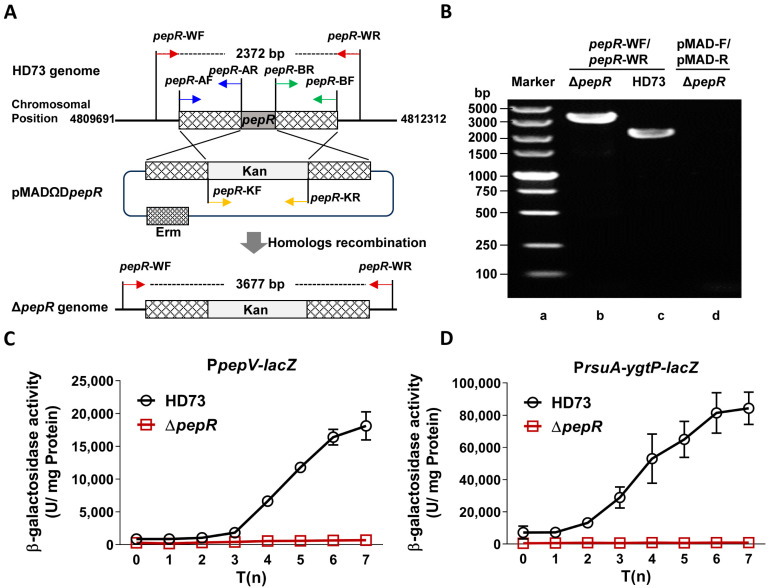
Transcription of *pepV* and *rsuA-ygtP* depended on *pepR*. (**A**) Construction of an in-frame deletion *pepR* mutant in HD73. The *pepR* gene was substituted by a kanamycin-resistant gene through double homolog recombination events (**B**) Identification of Δ*pepR* knockout mutant by PCR. PCR products were amplified from Δ*pepR* (Lane b) and HD73 (Lane c) with primer pair *pepR*-WF/*pepR*-WR. To confirm the presence of the pMADΩD*pepR* plasmid in the Δ*pepR* genome, PCR was performed using pMAD plasmid universal primers (pMAD-F/pMAD-R) (Lane d). The numbers indicate the size of the DNA standards in kilobase pairs (Lane a). (**C**) β-galactosidase activity of P*pepV*-*lacZ* in HD73 and Δ*pepR*. (**D**) β-galactosidase activity of P*rsuA-ygtP*-*lacZ* in HD73 and Δ*pepR*. The promoters of *pepV* and *rsuA-ygtP* were fused with the *lacZ* reporter and transformed into HD73 and Δ*pepR*, respectively. The β-galactosidase activities of three clones were determined at the indicated times after growing the cells in SSM at 30 °C. Each value represents the mean and standard error of at least three independent replicates.

To verify whether PepR regulates the transcription of *pepV* and *rsuA*-*ytgP*, we first constructed a *pepR* deletion mutant. The upstream (*pepR*-A, 1065 bp) and downstream (*pepR*-B, 1057 bp) homologous fragments of *pepR* were amplified from the HD73 genome DNA. Then, the *pepR*-A, *pepR*-B, and kanamycin resistance gene (*pepR*-K, 1506 bp) fragments were ligated together by overlapping PCR. The resulting 3563-bp fragment was inserted into the temperature-sensitive pMAD plasmid, leading to pMADΩD*pepR* plasmid. The pMADΩD*pepR* plasmid was electroporated into HD73. This transformant was then screened at 37 °C to identify the *pepR* deletion mutant, which lacked erythromycin resistance but was resistant to kanamycin. The diagram shows that the kanamycin resistance gene on the recombinant plasmid was exchanged by homolog recombination with *pepR* on the HD73 chromosome (Figure 3A). HD73 and Δ*pepR* strains were confirmed by PCR using primers of *pepR*-WF/*pepR*-WR. PCR products with the size of 3677 bp from Δ*pepR* and 2372 bp from HD73 were detected by agarose gel electrophoresis (Figure 3B). The Δ*pepR* DNA as template was amplified by PCR using primers (pMAD-F/pMAD-R) for detecting the pMAD plasmid, resulting in no PCR products (Figure 3B). It proved that the Δ*pepR* mutant was obtained.

Then, P*pepV* and P*rsuA*-*ytgP* promoter activity was measured in HD73 and ∆*pepR*. The P*pepV* and P*rsuA*-*ytgP* promoters were fused with the *lacZ* reporter gene and transformed into HD73 and ∆*pepR*, respectively. β-galactosidase activity showed that the activity of P*pepV* increased from T_0_ to T_7_ and reached the highest level at T_7_ in HD73. However, the transcriptional activity of P*pepV* was significantly reduced from T_0_ to T_7_ in ∆*pepR* (Figure 3C). The result revealed that the P*pepV* promoter is regulated by PepR. Similarly, the transcriptional activity of P*rsuA*-*ytgP* in the *pepR* mutant strain was also significantly decreased compared to that in HD73 from T_0_ to T_7_ (Figure 3D), which suggested that the *rsuA*-*ytgP* operon was also regulated by PepR.

### 3.4. PepR Binds to the pepV Promoter

**Figure 4:**
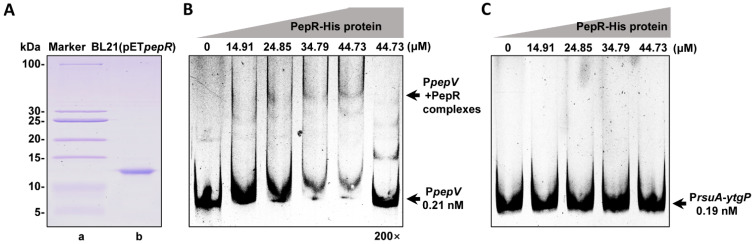
PepR directly bound *pepV* but not the *rsuA-ygtP* promoter region. (**A**) SDS-PAGE confirmed the expression and purification of the PepR-His protein by Ni^2+^-affinity chromatography. Lane a, standard proteins (PageRuler Prestained Protein Ladder 26632, Thermo, Rockford, IL, USA). Lane b, purified PepR from the BL21(pET*pepR*) strain. (**B**) EMSA detecting protein–DNA interactions using FAM-labeled P*pepV* and increasing concentrations of recombinant PepR. The lanes contained 0, 14.91, 24.85, 34.79, and 44.73 μM of the PepR protein. The last lane was employed for the 200-fold unlabeled probe. Protein–DNA complexes were separated by native-PAGE and FAM-labeled bands scanned using Typhoon9410 (Cytiva, Marlborough, MA, USA). (**C**) EMSA detecting protein–DNA interactions using FAM-labeled P*rusA*-*ytgP* and increasing concentrations of recombinant PepR.

To determine whether the PepR protein directly binds to the *pepV* or *rsuA*-*ytgP* promoter regions, the electrophoretic mobility shift assay (EMSA) was performed. We constructed the recombinant plasmid, pET*pepR*, which was able to express the His-tagged PepR protein and introduced it into the *E. coli* BL21(DE3). The PepR-His protein with a molecular weight of approximately 10.06 kDa was expressed in *E. coli* BL21(pET*pepR*) and purified by Ni^2+^-affinity chromatography (Figure 4A). Then, we tested the binding of the PepR protein to the *pepV* or *rsuA*-*ytgP* promoter regions. A 0.21 nM P*pepV*-labeled probe was used to bind to PepR (low concentrations of 34.79 μM). The P*pepV*-labeled probe at 0.21 nM was strongly shifted with a 44.73 nM PepR (Figure 4B). Notably, a 200-fold excess of unlabeled probe competed with the labeled probe, confirming the specific binding (Figure 4B). These results demonstrated that PepR directly binds to the *pepV* promoter region. However, when 0.19 nM of the P*rsuA*-*ytfP* probe was exposed to PepR concentrations ranging from 14.91 μM to 44.73 μM, no bind-shift could be detected, indicating that PepR cannot interact with the *rusA*-*ytfP* promoter region (Figure 4C). These results strongly support the theory that PepR directly regulates the expression of *pepV* and indirectly regulates *rusA*-*ytfP* expression.

### 3.5. ΔpepR and ΔpepV Mutants Are More Sensitive to Vancomycin Than HD73

**Figure 5:**
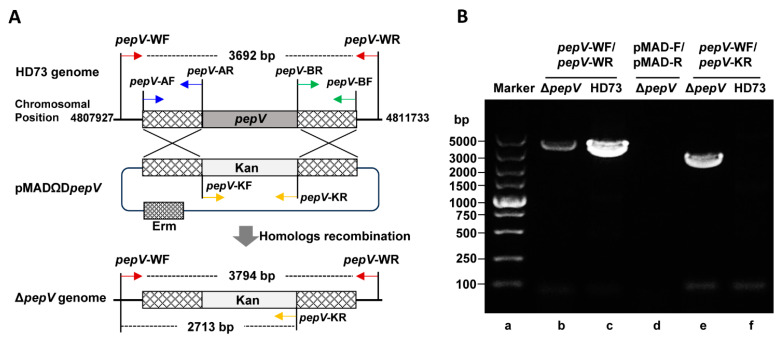
Construction of an in-frame deletion *pepV* mutant in HD73. (**A**) Construction of an in-frame deletion *pepV* mutant. The *pepV* gene was substituted by a kanamycin-resistant gene through double homolog recombination events. (**B**) Identification of the Δ*pepR* knockout mutant by PCR. PCR products were amplified from HD73 and Δ*pepV* strains using the primer pairs of *pepV*-WF/*pepV*-WR (Lanes b and c) and *pepV*-WF/*pepV*-KR (Lanes e and f). To confirm the presence of the pMADΩD*pepV* plasmid in the Δ*pepV* genome, PCR was performed using pMAD plasmid universal primers (pMAD-F/pMAD-R) (Lane d). The numbers indicate the size of the DNA standards in kilobase pairs (Lane a).

To characterize the function of the *pepV* gene, we constructed a *pepV* deletion mutant. The homolog arms on both sides of *pepV* were *pepV*-A (993 bp) and *pepV*-B (1023 bp) amplified from HD73 using primers *pepV-*AF/*pepV*-AR and *pepV-*BF/*pepV*-BR, respectively. The *pepV*-K fragment was obtained using PCR with *pepV*-KF/*pepV*-KR. The overlapping PCR products of *pepV*-A, *pepV*-B, and *pepV*-K were amplified with *pepV-*AF/*pepV-*BR primers, resulting in a 3452 bp fragment. The fragment was digested with BamHI and EcoRI sites and ligated to the temperature-sensitive vector of pMAD to obtain recombinant plasmid pMAD∆*pepV*. The recombinant plasmid pMAD∆*pepV* was electroporated into HD73. Transformants were selected for anti-sensitivity to erythromycin and kanamycin (Figure 5A). Positive transformants were verified at 37 °C. Colonies lacking erythromycin resistance and containing kanamycin resistance were selected for ∆*pepV*.

The *pepV* deletion mutant and HD73 strains were confirmed by PCR using *pepV*-WF/*pepV*-WR primers. PCR was performed with HD73 and ∆*pepV* chromosomal DNA as template. The product from ∆*pepV* was a 3794 bp fragment, while the product from HD73 was a 3692 bp fragment (Figure 5B, Lanes b and c). The Δ*pepV* mutant was confirmed using PCR with pMAD plasmid primers of pMAD-F/pMAD-R, which did not produce any PCR product (Figure 5B, Lane d). In addition, PCR products were generated using HD73 and ∆*pepV* chromosomal DNA as templates with primers (*pepV*-WF/*pepV*-KR). There was a 2713 bp fragment from ∆*pepV*, while no product from HD73 was obtained (Figure 5B, Lanes f and e). The result confirms that the Δ*pepV* mutant was successfully obtained.

**Figure 6:**
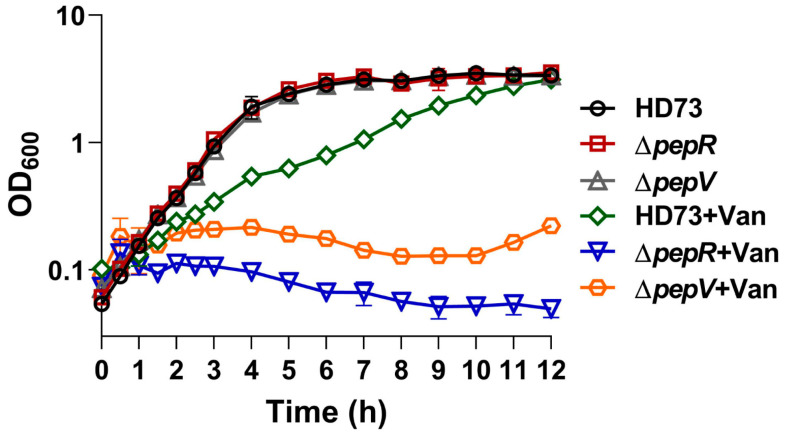
∆*pepR* and Δ*pepV* mutant strains were more sensitive to vancomycin compared to HD73. Bacteria growth was monitored every hour after inoculation to culture medium with vancomycin at 6 ng/mL. Growth curves represent means of three clones.

It has been demonstrated that PepV is related to vancomycin resistance in *L. lactis* MG1363 [29]. A vancomycin resistance test was performed by inoculating ∆*pepR*, ∆*pepV*, and HD73 in SSM medium with 6 ng/mL concentrations of vancomycin. The result showed that the absence of vancomycin had no effect on the growth of ∆*pepR*, ∆*pepV*, and HD73 strains. However, the addition of vancomycin slowed HD73 growth. ∆*pepV* growth stagnated while ∆*pepR* lysed and died after vancomycin was added (Figure 6). The result suggests that ∆*pepR* and ∆*pepV* were more sensitive to vancomycin than HD73, and ∆*pepR* was more sensitive to vancomycin than ∆*pepV*.

## 4. Discussion

In our previous study, we validated the strong promoter activity from the *pepR* gene which encodes a DeoR family transcriptional regulator [12]. The *pepR* promoter is activated by Sigma E and can direct *cry1Ac* expression efficiently [12]. However, the functions of the *pepR* gene and its targets were unknown. In this study, we found that PepR homologs are widely distributed in various Gram-positive bacteria, and the amino acid similarities are highly conserved in different bacteria (Figure 1). We selected 18 strains that have been extensively studied for exhaustive analyses of the *pepR* genetic loci. We found that *pepV*, *pepR* and *rusA*-*ytgP* were closely linked in some strains, such as *B. thuringiensis* HD73, *B. cereus* ATCC 14579, *B. cytotoxicus*, *B. anthracis* Ames, *Planococcus antarcticus*, *Ectobacillus* sp. JY-23, *Sporosarcina psychrophile*, and *Sutcliffiella horikoshii*, while in some other strains they were not closely linked, such as in *Anoxybacillus caldiproteolyticus*, *A. flavithermus*, *Priestia koreensis*, *Mangrovibacillus cuniculi*, *P. megaterium* QM B1551, *B. pumilus*, *Lysinibacillus fusiformis*, *Aeribacillus pallidus* KCTC 3564, *Metabacillus sediminilitoris*, and *Cytobacillus spongiae* (Figure S1). In HD73, *pepV* and *rusA*-*ytgP* were two independent operons, and their promoters failed to transcript in the *pepR* knockout strain (Figure 2 and Figure 3), suggesting these genes are all controlled by transcriptional regulator PepR. We further found that PepR directly binds to the *pepV* promoter but not to the *rusA*-*ytgP* promoter (Figure 4 and Figure 5), revealing that *pepV* acts as a downstream target of *pepR*. The *pepV* promoter regions were 98.95% similar in different *B. thuringiensis* strains, and we determined that the expression of *pepV* is regulated by PepR. Whether PepR homologs also regulate *pepV* transcription in other bacteria remains to be confirmed. In addition, other targets of PepR should be investigated using RNA-seq and ChIP-seq methods in following studies.

PepV is highly conserved within the dipeptidase M20 family in the metallopeptidases [30]. It has been reported that the active sites and conserved domains of all PepVs are similar [30,31,32]. We analyzed the amino acid sequence of the PepV homologs and found that the identity varies in *B. subtilis* (57.36%), *L. delbrueckii* (34.62%), *L. helveticus* (33.12%), *S. gordonii* (40.86%) and *L. lactis* (38.92%) compared to HD73 (Figure S2). This result suggests that PepV in HD73 may have a similar function in catalyzing substrates to that reported in other bacteria. It is worth mentioning that the deletion of *pepV* in HD73 increased the susceptibility to vancomycin, whereas the deletion of *pepV* in *L. lactis* MG1363 decreased the susceptibility of *L. lactis* to vancomycin [29]. In addition to BCARR regulating *pepV* expression in the presence of BCAAs in *L. helveticus* [17] and a CodY-like regulatory system controlling the expression of *pepV* in *L. helveticus* CM4 [16], *pepV* might have multiple functions in responding to different signals in different bacteria strains.

## Figures and Tables

**Table 1:** Strains and plasmids used in this study.

Strain or Plasmid	Relevant Details ^a^	Reference or Source
*E. coli* strains		
*E. coli* TG1	∆(*lac-proAB*) *supE thi hsd-5* (*F′ traD36 proA^+^ proB^+^ lacI^q^ lacZ*∆M15), general purpose cloning host	Laboratory collection
*E. coli* ET 12567	*F^−^ dam-13*::Tn 9 *dcm-6 hsdM hsdR recF143 zjj-202*::Tn10 *galK2 galT22 ara14 pacY1 xyl-5 leuB6 thi-1*, for generation of unmethylated DNA	Laboratory collection
BL21 (DE3)	*F^−^ dcmopmT hsds* (r_B_*^−^* m_B_*^−^*) galλ(DE3)	[21]
BL21 (pET*pepR*)	BL21(DE3) with pET*pepR* plasmid	This study
*B. thuringiensis* strains		
HD73	Wild-type strain containing plasmid pHT73 carrying *cry1Ac* gene	Laboratory collection
∆*pepR*	HD73 mutant, *pepR* gene was deleted by homologs recombination	This study
∆*pepV*	HD73 mutant, *pepV* gene was deleted by homologs recombination	This study
HD (P*pepV*)	HD73 strain containing plasmid pHT304-P*pepV*-18Z	This study
*∆pepR* (P*pepV*)	*pepR* mutant containing plasmid pHT304-P*pepV*-18Z	This study
HD (P*rsuA*-*ytgP*)	HD73 strain containing plasmid pHT304- P*rsuA*-*ytgP*-18Z	This study
*∆pepR* (P*rsuA*-*ytgP*)	*pepR* mutant containing plasmid pHT304- P*rsuA*-*ytgP*-18Z	This study
Plasmids		
pHT304-18Z	*E. coli*-Bt shuttle vector with promoter-less *lacZ* reporter, Amp^R^, Erm^R^	[22]
pET-21b	Expression vector; Amp^r^ 5.4 kb	Laboratory collection
pMAD	Amp^R^, Erm^R^, temperature-sensitive *E. coli*-*B. thuringiensis* shuttle vector	[23]
pMADΩD*spo0A*	pMAD with *spo0A* deletion fragment, Amp^R^, Erm^R^, Kan^R^	[24]
pMADΩD*pepR*	pMAD with *pepR* deletion fragment, Amp^R^, Erm^R^, Kan^R^	This study
pMADΩD*pepV*	pMAD with *pepV* deletion fragment, Amp^R^, Erm^R^, Kan^R^	This study
pET*pepR*	pET-21b containing *pepR* gene; Amp^r^	This study
304P*pepV*	pHT304-18Z carrying P*pepV*, Amp^R^, Erm^R^	This study
304P*rsuA*-*ytgP*	pHT304-18Z carrying P*rsuA*-*ytgP*, Amp^R^, Erm^R^	This study

^a^ Antibiotic resistance cassettes are indicated as follows: Erm^R^, erythromycin resistance; Kan^R^, kanamycin resistance; Amp^R^, ampicillin resistance.

**Table 2:** Oligonucleotide primers used in this study.

Primer Name	Sequence (5′–3′) ^a^	Restriction Site
pET*pepR*-F	CGGGATCCGTTGAAACCTACAACTACTCG	BamHI
pET*pepR*-R	ACGCGTCGACTGAGGTCATTCTCACTTTC	SalI
*pepV*-AF	GTACCCGGGAGCTCGAATTCCGAAATGTCCGACTTGTTCCATACG	EcoRI
*pepV*-AR	TCACCTCAAATGGTTCGCTGGAATTAGCGAAGTAATGGATATATAAATAATGTCCGCTC	
*pepV*-KF	GAGCGGACATTATTTATATATCCATTACTTCGCTAATTCCAGCGAACCATTTGAGGTGA	
*pepV*-KR	GAAGGATGGATGCGTGATGTCAGCAATTAATTGGAAATTCCTCGTAGGCGCTCG	
*pepV*-BF	CGAGCGCCTACGAGGAATTTCCAATTAATTGCTGACATCACGCATCCATCCTTC	
*pepV*-BR	CGTCGGGCGATATCGGATCCGCACACGTTGCAGGAGTAGTAACAGAAG	BamHI
*pepV*-WF	GATACACAGCACCTAAATCTGTACCTTCG	
*pepV*-WR	CCCAGTTGGACGACTTGATATCGATACGGAAGG	
*pepR*-AF	GTACCCGGGAGCTCGAATTCAATCAAATGAAACAAGTTCAT	EcoRI
*pepR*-AR	TCAAATGGTTCGCTGAATGCGTGTTAGCATACGAG	
*pepR*-KF	ATGCTAACACGCATTCAGCGAACCATTTGAGGTGA	
*pepR*-KR	TTATGAGGTCATTCTAAATTCCTCGTAGGCGCTCG	
*pepR*-BF	GCCTACGAGGAATTTAGAATGACCTCATAATGAAA	
*pepR*-BR	CGTCGGGCGATATCGGATCCCAATTTGTGCAGGTATTGGC	BamHI
*pepR*-WF	CTTCTCCTGTAATAACCGCTTCTGC	
*pepR*-WR	CTAACACTTATAATGGTCGTTGCTG	
pMAD-F	CCAAATTTCCTCTGGCCATT	
pMAD-R	CCTATACCTTGTCTGCCTCC	
P*pepV*-F	CGCGGATCCCATCACGCATCCATCCTTCACT	BamHI
P*pepV*-R	AACTGCAGGAGAAAACCACTCCTCTAC	PstI
P*rsuA*-*ytgP*-F	AACTGCAGCTGCAGCACCAATTGCAGCCATTAG	PstI
P*rsuA*-*ytgP*-R	CGCGGATCCGTAACAATAAGCGTTCCGCGCAG	BamHI
RT-*thpR*-F	ACGAGCAACGGTAATA	
RT-*thpR*-R	GGGCGAAGGTAAGTGA	
RT-*pepV*-F	TGATGTGGGCGAGAAT	
RT-*pepV*-R	ACGGTGGTAACTTAGGG	
RT-*thpR*-*pepV*-F	GTACATCTTCTCCTTCTTTC	
RT-*thpR*-*pepV*-R	GGCCCGCTATTCCCTGG	
RT-*rsuA*-F	CATCCTCTTCTGTTACTACTCC	
RT-*rsuA*-R	CAGCGACGGAAGATGATAATC	
RT-*ytgP*-F	GCTCCTACTGTACCGAAATAACG	
RT-*ytgP*-R	GATTCAGATCCACTAGGTGGAC	
RT-*rsuA*-*ytgP*-F	GTTTTTCACCTTCTTTATTTATC	
RT-*rsuA*-*ytgP*-R	CATGATTTCGCCTGATGGCCG	
RT-*16S*-F	TCGCATTAGCTAGTTGGTGAG	
RT-*16S*-R	TCTTCCCTAACAACAGAGTTT	

^a^ Restriction enzyme sites are underlined.

## Data Availability

Data are contained within the article.

## Supplementary Materials

The following supporting information can be downloaded at: https://www.mdpi.com/article/10.3390/microorganisms12030579/s1, Figure S1: The *pepR* gene cluster is a conserved in bacteria; Figure S2: Comparison of the amino acid sequences revealed a conserved PepV homologs in bacteria; Table S1: List of species in Figure 1.
