# Supplementary material for: A Novel Regulator PepR Regulates the Expression of Dipeptidase Gene pepV in Bacillus thuringiensis
Source: Microorganisms. 2024 Mar 14;12(3):579. doi: 10.3390/microorganisms12030579 (PMC10975445; doi:10.3390/microorganisms12030579)
Supplement: Supplementary file 1 [file microorganisms-12-00579-s001.zip › microorganisms-2907715-supplementary.pdf]

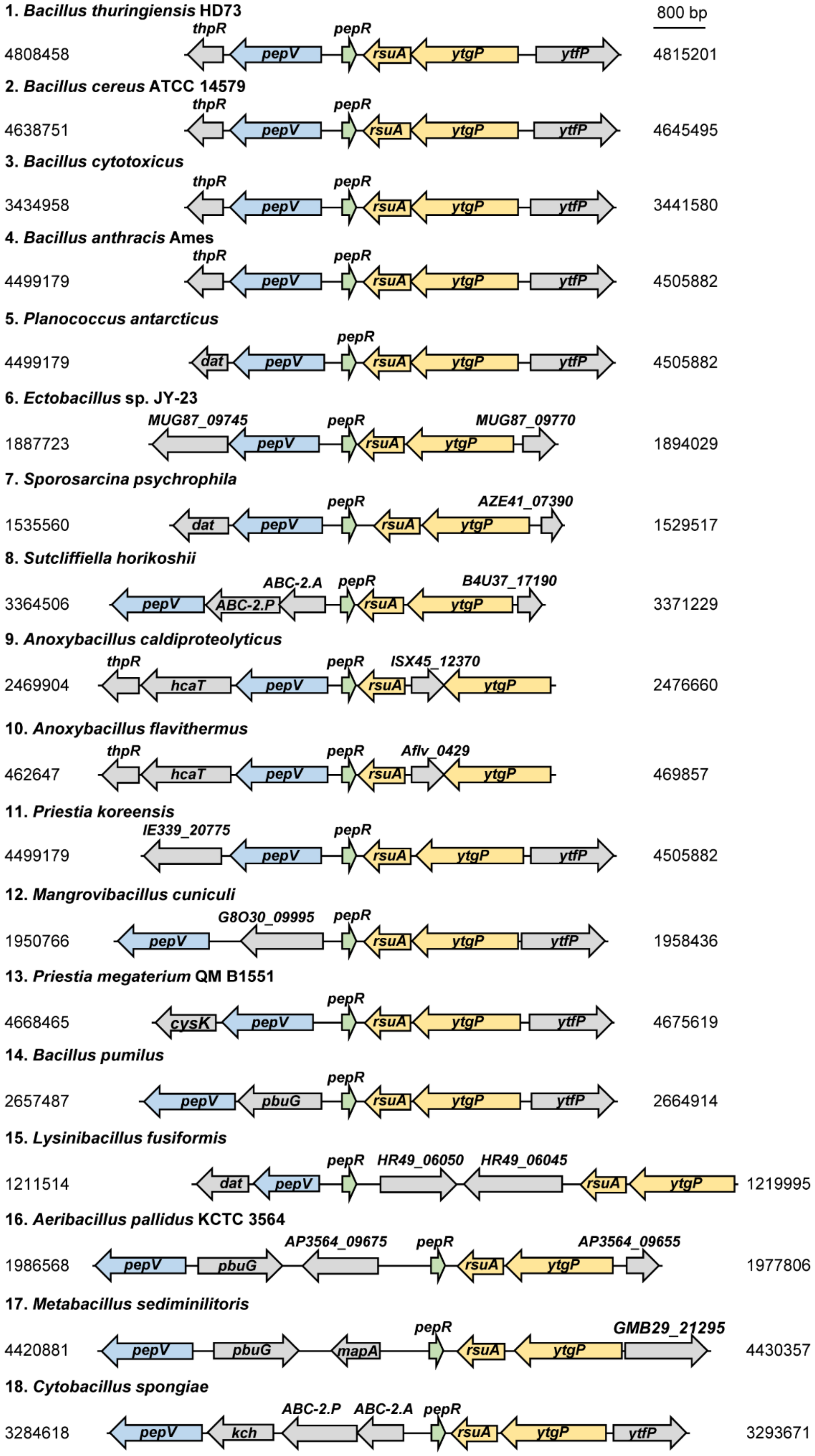
1. Bacillus thuringiensis HD73
800 bp
4808458
thpR
pepV
pepR
rsuA
ytgP
ytfP
4815201
2. Bacillus cereus ATCC 14579
4638751
4645495
3. Bacillus cytotoxicus
3434958
3441580
4. Bacillus anthracis Ames
4499179
4505882
5. Planococcus antarcticus
dat
4499179
4505882
6. Ectobacillus sp. JY-23
MUG87_09745
MUG87_09770
1887723
1894029
7. Sporosarcina psychrophila
AZE41_07390
1535560
1529517
8. Sutcliffiella horikoshii
ABC-2.A
ABC-2.P
B4U37_17190
3364506
3371229
9. Anoxybacillus caldiproteolyticus
hcaT
ISX45_12370
2469904
2476660
10. Anoxybacillus flavithermus
Aflv_0429
462647
469857
11. Priestia koreensis
IE339_20775
4499179
4505882
12. Mangrovibacillus cuniculi
G8O30_09995
1950766
1958436
13. Priestia megaterium QM B1551
cysK
4668465
4675619
14. Bacillus pumilus
pbuG
2657487
2664914
15. Lysinibacillus fusiformis
HR49_06050
HR49_06045
1211514
1219995
16. Aeribacillus pallidus KCTC 3564
AP3564_09675
AP3564_09655
1986568
1977806
17. Metabacillus sediminilitoris
mapA
GMB29_21295
4420881
4430357
18. Cytobacillus spongiae
kch
3284618
3293671

Figure S1. The *pepR* gene cluster is a conserved in bacteria. The neighboring loci of *pepR* in the genome were *thpR* (RNA 2',3'-cyclic phosphodiesterase), *pepV* (dipeptidase), *rsuA* (rRNA pseudouridine synthase), *ytgP* (polysaccharide biosynthesis protein), and *ytfP* (NAD(P)/FAD-dependent oxidoreductase). Blue, green, and yellow arrows indicate *pepV, pepR*, and *rsuA*-*ytgP* operon, respectively. The numbers at the ends of the lines represent the locations in the genome for the two end bases.

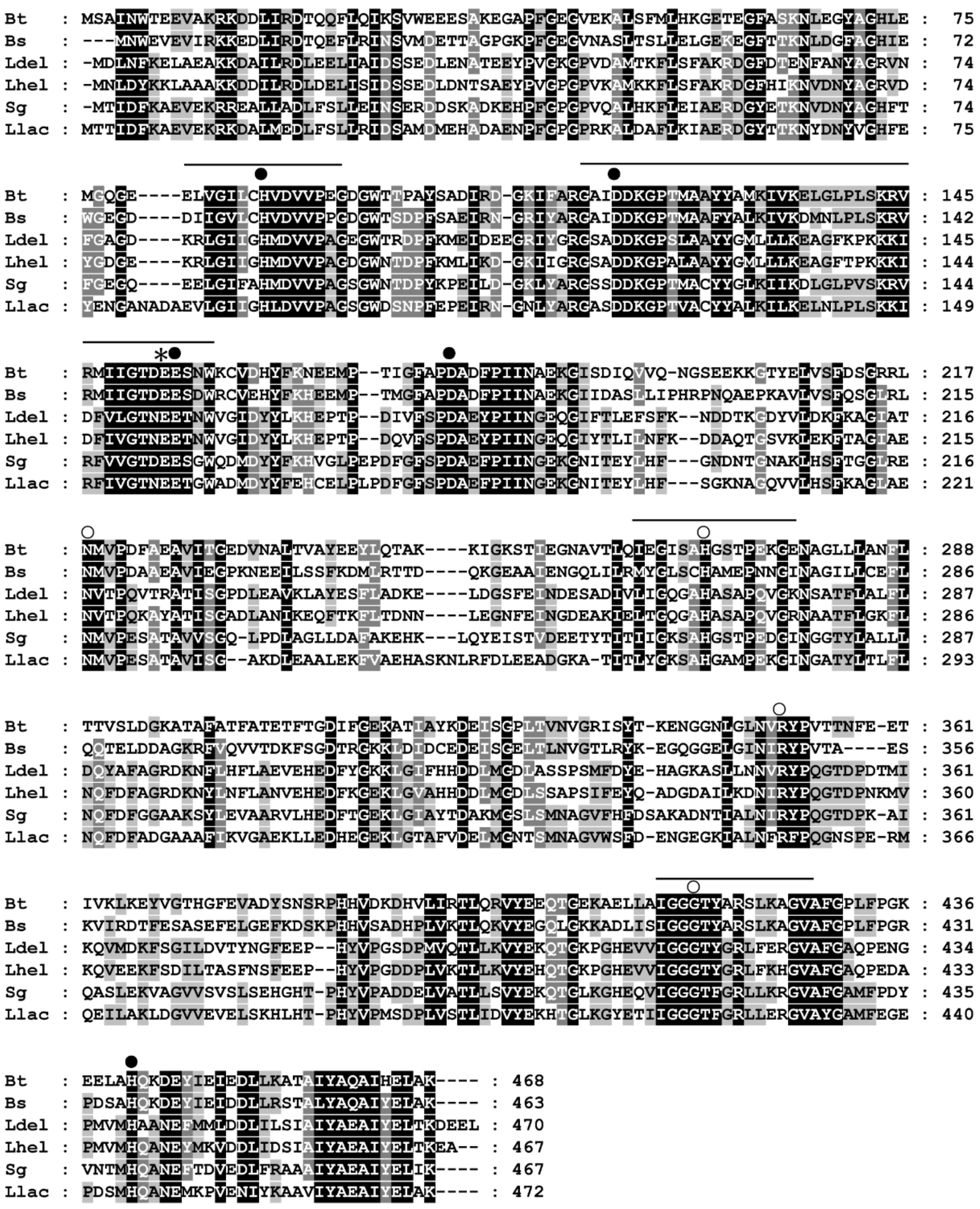

Figure S2. Comparison of the amino acid sequences revealed a conserved PepV homologs in bacteria. Sequences from *Bacillus thuringiensis* HD73 (Bt) [WP_001274251.1], *Bacillus subtilis* 168 (Bs) [WP_004399126.1], *Lactobacillus delbrueckii subsp. Lactis* DSM7290 (Ldel) [WP_014565245.1], *Lactobacillus helveticus* SBT2171 (Lhel) [WP_003626366.1], *Lactococcus lactis* MG1363 (Llac) [WP_011835507.1] and *Streptococcus gordonii* (Sg) [VTT24557.1] were aligned with the ClustalW multiple sequence alignment tool according to homology modeling

(grey shading indicates similarity, and black shading indicates identity). Conserved box regions (boxes 1 to 4) are in sequential order and are denoted with single lines. An asterisk (*) indicates the catalytic site, closed circles (●) indicate the zinc-binding sites, and open circles (○) indicate the substrate-binding sites.

Supplementary Table S1. List of species in Figure 1

Species	Accession	Cover	E-value	Identity
Bacillus thuringiensis HD73	WP_000805512.1	100%	2.07E-45	100%
Bacillus thuringiensis	HDX9497500.1	100%	6.55E-45	98.63%
Bacillus cereus	HDW3053053.1	100%	7.63E-45	98.63%
Bacillus cytotoxicus	MCM3734429.1	100%	1.52E-44	98.63%
Bacillus anthracis	WP_047398659.1	100%	5.04E-43	98.63%
Ectobacillus funiculus	WP_129727866.1	100%	1.10E-38	86.30%
Domibacillus indicus	WP_046176652.1	100%	8.47E-38	86.30%
Ectobacillus panaciterrae	WP_028399761.1	100%	2.74E-37	84.93%
Ectobacillus	WP_124564370.1	100%	4.40E-37	86.30%
Domibacillus antri	WP_075399267.1	100%	4.50E-37	83.56%
Domibacillus epiphyticus	WP_076764548.1	100%	8.04E-37	84.93%
Thermolongibacillus altinsuensis	WP_132949297.1	100%	3.21E-36	79.45%
Bacillus kexueae	WP_243385999.1	100%	7.56E-36	78.08%
Kurthia massiliensis	WP_010288356.1	100%	8.25E-36	82.19%
Rossellomorea aquimaris	WP_113969813.1	100%	1.41E-35	79.45%
Aeribacillus pallidus	MDR9796532.1	100%	1.92E-35	76.71%
Bacillus salacetis	WP_119546235.1	100%	1.99E-35	80.82%
Bacillus xiapuensis	WP_100331890.1	100%	2.17E-35	79.45%
Anoxybacillus tepidamans	WP_084279132.1	100%	2.34E-35	78.08%
Gottfriedia endophyticus	WP_209406692.1	100%	2.82E-35	78.08%
Heyndrickxia sporothermodurans	GIN88161.1	100%	3.08E-35	79.45%
Metabacillus litoralis	WP_226670209.1	100%	3.18E-35	78.08%
Heyndrickxia oleronia	WP_078111183.1	100%	3.26E-35	79.45%
Anoxybacillus caldiproteolyticus	WP_181554765.1	100%	3.44E-35	79.45%
Mesobacillus maritimus	WP_251431821.1	100%	4.38E-35	79.45%
Ectobacillus ponti	WP_254757185.1	100%	4.89E-35	80.82%
Planococcus sp. ISL-109	WP_215106009.1	100%	6.02E-35	83.56%
Sutcliffiella rhizosphaerae	WP_230502367.1	100%	7.75E-35	76.71%
Rummeliibacillus pycnus	WP_102691013.1	100%	7.75E-35	80.82%
Metabacillus sediminilitoris	WP_136352094.1	100%	7.92E-35	78.08%
Bacillus weihaiensis	WP_072578818.1	100%	9.55E-35	78.08%
Bacillus ectoiniformans	WP_204556568.1	100%	9.76E-35	78.08%
Anoxybacillus vitaminiphilus	WP_111644448.1	100%	1.04E-34	78.08%
Kurthia sibirica	WP_109305283.1	100%	1.04E-34	80.82%
Paenisporosarcina indica	WP_075620108.1	100%	1.11E-34	76.71%
Sutcliffiella horikoshii	WP_063559844.1	100%	1.26E-34	76.71%
Psychrobacillus sp. OK028	WP_093060335.1	100%	1.28E-34	79.45%
Psychrobacillus insolitus	WP_111437610.1	100%	1.36E-34	79.45%
Candidatus Kurthia equi	MBQ0139077.1	100%	1.55E-34	80.82%

Species	Accession	Cover	E-value	Identity
Psychrobacillus sp. OK032	WP_093267179.1	100%	1.73E-34	79.45%
Mangrovibacillus cuniculi	WP_239671949.1	100%	1.97E-34	76.71%
Bacillus coahuilensis	WP_010174203.1	100%	2.40E-34	76.71%
Mesobacillus subterraneus	WP_125479558.1	100%	2.48E-34	79.45%
Mesobacillus persicus	WP_090741696.1	100%	2.71E-34	78.08%
Anoxybacillus calidus	WP_181537608.1	100%	3.13E-34	76.71%
Planococcaceae	WP_084246586.1	100%	3.16E-34	82.19%
Metabacillus iocasae	WP_205188859.1	100%	3.20E-34	78.08%
Bacillus alveayuensis	WP_044894459.1	100%	3.27E-34	76.71%
Bacillus massiliglaciei	WP_110927274.1	100%	5.59E-34	75.34%
Jeotgalibacillus proteolyticus	WP_104057185.1	100%	5.87E-34	79.17%
Jeotgalibacillus salarius	WP_134382991.1	100%	6.21E-34	79.17%
Oikeobacillus pervagus	WP_307257751.1	100%	6.38E-34	75.34%
Psychrobacillus psychrodurans	WP_090564512.1	100%	6.89E-34	78.08%
Bacillota bacterium	MBU0905012.1	100%	8.13E-34	75.34%
Psychrobacillus vulpis	WP_142643845.1	100%	8.22E-34	78.08%
Priestia koreensis	WP_242170838.1	100%	8.97E-34	75.34%
Planococcus sp. MER TA 32b	MCM3611524.1	100%	9.69E-34	80.82%
Metabacillus arenae	WP_191157570.1	100%	1.12E-33	78.08%
Bacillus massiliigorillae	WP_042345227.1	100%	1.17E-33	75.34%
Peribacillus glennii	WP_117320796.1	100%	1.50E-33	75.34%
Litchfieldia salsa	WP_090849283.1	100%	1.54E-33	76.71%
Terrabacteria group	WP_142829443.1	100%	1.55E-33	80.82%
Metabacillus fastidiosus	WP_066234314.1	100%	1.83E-33	72.60%
Lederbergia panacisoli	WP_257581349.1	100%	1.91E-33	75.34%
Planococcus salinus	WP_123163543.1	100%	2.02E-33	80.82%
Mesobacillus foraminis	WP_121611380.1	100%	2.07E-33	76.71%
Chryseomicrobium aureum	WP_204590502.1	100%	2.11E-33	76.71%
Falsibacillus pallidus	WP_114745200.1	100%	2.36E-33	72.60%
Sutcliffiella halmapala	WP_078379340.1	100%	2.38E-33	75.34%
Anoxybacillus flavithermus	WP_004888976.1	100%	2.87E-33	72.60%
Psychrobacillus glaciei	WP_151700651.1	100%	2.94E-33	76.71%
Geobacillus	WP_023817707.1	100%	3.58E-33	76.71%
Bacillus chungangensis	WP_307230403.1	100%	3.70E-33	75.34%
Cytobacillus spongiae	WP_233807561.1	100%	3.99E-33	75.34%
Bacillus massilionigeriensis	WP_075982639.1	100%	4.26E-33	73.97%
Bacillus sinesaloumensis	WP_077619127.1	100%	4.29E-33	76.39%
Geobacillus proteiniphilus	WP_307898757.1	100%	4.56E-33	76.71%
Bacillus thermotolerans	WP_039234094.1	100%	4.66E-33	75.34%

Species	Accession	Cover	E-value	Identity
Priestia megaterium	WP_098658829.1	100%	4.76E-33	76.71%
Xanthomonas citri	MBD5023001.1	100%	5.14E-33	75.34%
Litchfieldia alkalitelluris	WP_078544971.1	100%	5.31E-33	73.97%
Bacillus pumilus	WP_119125252.1	100%	5.55E-33	73.97%
Bacillus aquiflavi	WP_163242368.1	100%	5.55E-33	73.97%
Peribacillus loiseleuriae	WP_049682664.1	100%	5.86E-33	73.97%
Paenisporosarcina sp. HGH0030	WP_016426574.1	100%	5.86E-33	73.97%
Bacillus amyloliquefaciens group	WP_165882092.1	100%	6.26E-33	73.97%
Peribacillus asahii	WP_119116910.1	100%	6.26E-33	72.60%
Bacillus kwashiorkori	WP_062354866.1	100%	6.54E-33	73.97%
Cytobacillus purgationiresistens	WP_307477527.1	100%	6.76E-33	73.97%
Bacillus haynesii	WP_043926249.1	100%	6.99E-33	73.97%
Heyndrickxia shackletonii	WP_055738983.1	100%	7.63E-33	75.34%
Ureibacillus thermophilus	WP_208650260.1	100%	8.52E-33	75.34%
Bacillus amyloliquefaciens	WP_289393838.1	100%	9.00E-33	73.97%
Cytobacillus horneckiae	WP_066192053.1	100%	9.61E-33	73.97%
Fredinandcohnia sp. SECRCQ15	WP_240254714.1	100%	9.93E-33	75.34%
Metabacillus kandeliae	WP_231787338.1	100%	1.12E-32	76.71%
Lysinibacillus fusiformis	QDP99819.1	100%	1.15E-32	75.34%
Paenisporosarcina quisquiliarum	WP_269925438.1	100%	1.18E-32	73.97%
Robertmurraya massiliosenegalensis	WP_019154908.1	100%	1.25E-32	73.97%
Planococcus antarcticus	WP_006830850.1	100%	1.31E-32	78.08%
Neobacillus sp. PS3-12	WP_308174508.1	100%	1.31E-32	75.34%
Neobacillus pocheonensis	MCM2531377.1	100%	1.72E-32	76.71%
Parageobacillus thermantarcticus	WP_090947684.1	100%	1.86E-32	73.97%
Neobacillus kokaensis	WP_191270063.1	100%	2.05E-32	75.34%
Heyndrickxia camelliae	WP_101354979.1	100%	2.26E-32	73.97%
Heyndrickxia acidicola	WP_066270043.1	100%	3.32E-32	73.97%
Neobacillus mesonae	WP_251471347.1	100%	3.40E-32	75.34%
Peribacillus deserti	WP_101643144.1	100%	4.09E-32	73.97%
Neobacillus vireti	WP_024026706.1	100%	4.09E-32	75.34%
Neobacillus piezotolerans	WP_115452643.1	100%	4.14E-32	75.34%
Bacillus suaedaesalsae	WP_204201773.1	100%	5.57E-32	73.97%
Neobacillus soli	WP_084374900.1	100%	5.88E-32	73.97%
Bacillus litorisediminis	WP_243298092.1	100%	6.42E-32	76.71%
Edaphobacillus lindanitolerans	WP_076758254.1	100%	7.17E-32	75.34%
Neobacillus dielmonensis	WP_042459807.1	100%	7.33E-32	73.97%
Neobacillus terrae	WP_081680106.1	100%	7.33E-32	75.34%
Siminovitchia thermophila	WP_077111916.1	100%	8.27E-32	72.60%

Species	Accession	Cover	E-value	Identity
Sporosarcina psychrophila	HJF31552.1	100%	9.53E-32	73.97%
Lysinibacillus contaminans	WP_053582402.1	100%	1.05E-31	72.60%
Robertmurraya kyonggiensis	WP_136832819.1	100%	1.06E-31	72.60%
Peribacillus psychrosaccharolyticus	WP_040372415.1	100%	1.08E-31	71.23%
Bacillus renqingensis	WP_199421031.1	100%	1.09E-31	73.97%
Caldibacillus debilis	WP_020156918.1	100%	1.33E-31	73.97%
Lederbergia lenta	WP_066145518.1	100%	1.35E-31	72.60%
Bacillus benzoevorans	WP_184526108.1	100%	1.78E-31	73.97%
Peribacillus tepidiphilus	WP_153127386.1	100%	1.90E-31	72.60%
Sporosarcina sp. ANT_H38	WP_149578880.1	100%	2.01E-31	72.60%
Bacillus sp. FJAT-29937	WP_066294579.1	100%	2.20E-31	72.60%
Lysinibacillus sphaericus	WP_024363739.1	100%	2.24E-31	72.60%
Sporosarcina limicola	WP_192598170.1	100%	2.71E-31	71.23%
Rossellomorea vietnamensis	WP_148945767.1	100%	3.05E-31	76.71%
Neobacillus fumarioli	WP_066366879.1	100%	3.05E-31	72.60%
Sporosarcina sp. ACRSM	WP_239390431.1	100%	3.22E-31	71.23%
Bacillus yapensis	WP_126409524.1	100%	3.41E-31	71.23%
Lysinibacillus alkalisoli	WP_188615505.1	100%	3.89E-31	72.60%
Bhargavaea cecembensis	WP_008300161.1	100%	4.43E-31	71.23%
Weizmannia acidilactici	WP_151679414.1	100%	4.89E-31	75.34%
Bhargavaea beijingensis	WP_092097684.1	100%	5.52E-31	71.23%
Caldibacillus thermoamylovorans	WP_034771864.1	100%	6.73E-31	75.34%
Bacillus dakarensis	WP_077214294.1	100%	6.73E-31	72.60%
Heyndrickxia acidiproducens	WP_018664029.1	100%	6.73E-31	75.34%
Heyndrickxia ginsengihumi	WP_025729975.1	100%	7.35E-31	75.34%
Bacillus oleivorans	WP_097158925.1	100%	9.25E-31	73.97%
